# Jet Cutting Technique for the Production of Chitosan Aerogel Microparticles Loaded with Vancomycin

**DOI:** 10.3390/polym12020273

**Published:** 2020-01-29

**Authors:** Clara López-Iglesias, Joana Barros, Inés Ardao, Pavel Gurikov, Fernando J. Monteiro, Irina Smirnova, Carmen Alvarez-Lorenzo, Carlos A. García-González

**Affiliations:** 1 Department of Pharmacology, Pharmacy and Pharmaceutical Technology, I+D Farma group (GI-1645), Faculty of Pharmacy, Agrupación Estratégica de Materiales (AeMAT) and Health Research Institute of Santiago de Compostela (IDIS), Universidade de Santiago de Compostela, E-15782 Santiago de Compostela, Spain; clara.lopez.iglesias@rai.usc.es (C.L.-I.); carmen.alvarez.lorenzo@usc.es (C.A.-L.); 2 Instituto de Investigação e Inovação em Saúde (i3S), Instituto Nacional de Engenharia Biomédica (INEB) and Faculdade de Engenharia Universidade do Porto (FEUP), Universidade do Porto, 4200-135 Porto, Portugal; joanabarros@fe.up.pt (J.B.); fjmont@fe.up.pt (F.J.M.); 3 BioFarma Research group, Center for Research in Molecular Medicine and Chronic Diseases (CiMUS), Universidade de Santiago de Compostela, E-15782 Santiago de Compostela, Spain; ines.ardao@usc.es; 4 Laboratory for Development and Modelling of Novel Nanoporous Materials, Eißendorfer Str. 38, 21073 Hamburg, Germany; pavel.gurikov@tuhh.de; 5 Institute of Thermal Separation Processes, Hamburg University of Technology, Eißendorfer Str. 38, 21073 Hamburg, Germany; irina.smirnova@tuhh.de

**Keywords:** biopolymers, polymer processing, biomedical applications, wound treatment, chitosan, aerogels

## Abstract

Biopolymer-based aerogels can be obtained by supercritical drying of wet gels and endowed with outstanding properties for biomedical applications. Namely, polysaccharide-based aerogels in the form of microparticles are of special interest for wound treatment and can also be loaded with bioactive agents to improve the healing process. However, the production of the precursor gel may be limited by the viscosity of the polysaccharide initial solution. The jet cutting technique is regarded as a suitable processing technique to overcome this problem. In this work, the technological combination of jet cutting and supercritical drying of gels was assessed to produce chitosan aerogel microparticles loaded with vancomycin HCl (antimicrobial agent) for wound healing purposes. The resulting aerogel formulation was evaluated in terms of morphology, textural properties, drug loading, and release profile. Aerogels were also tested for wound application in terms of exudate sorption capacity, antimicrobial activity, hemocompatibility, and cytocompatibility. Overall, the microparticles had excellent textural properties, absorbed high amounts of exudate, and controlled the release of vancomycin HCl, providing sustained antimicrobial activity.

## 1. Introduction

Aerogels are nanostructured, lightweight materials with open, high porosities and large surface areas that currently find applications in many industrial sectors due to their thermal, optical, electrical, or mechanical properties [1,2]. The outstanding textural properties of the aerogels have also attracted the attention from other fields such as biomedical and environmental sciences [3,4,5,6]. Biomedical applications of aerogels include the encapsulation of bioactive agents with solubility or stability limitations, and their use as synthetic scaffolds for tissue engineering and wound dressing materials for chronic wounds [7,8,9,10,11,12,13,14,15,16]. In the latter case, the large surface area of the aerogels confers them the ability to load and release bioactive agents, such as antibiotics or growth factors, which can facilitate the wound healing process [17]. High porosity also provides the aerogels with the ability to absorb exudate fluid in order to maintain a correct moisture balance at the wound site. This in turn alleviates the inflammatory process and prevents the appearance of bacterial infections (one of the main barriers for the wound healing process) [11,18]. Other advantages of aerogels include high stability upon storage and protection of the drug from the environment [5].

Aerogels are usually processed by supercritical drying of polymeric wet gels with supercritical CO_2_ (scCO_2_) [19]. This drying technique can extract the liquid part of the gel under mild conditions of pressure and temperature (P > 73.8 bar, T > 31.1 °C) whilst preserving the interconnected structure of the polymer network without causing the pore collapse phenomenon. Biopolymer-based materials are preferred for wound care applications, since their biocompatibility and biodegradability may avoid toxicity problems. Natural polysaccharides such as alginate, pectin, cellulose, starch, or chitosan are widely used since they are abundant and cost-effective, and have been approved and largely used in the food and pharmaceutical industry [20,21,22]. The hemostatic, antimicrobial, and healing-promoting properties of chitosan are suitable for wound healing applications [23,24]. Chitosan gels can be processed by physical or chemical crosslinking, resulting in tunable mechanical properties and enhanced biocompatibility [25].

Particulate systems, such as micro- or nanoparticles, are attracting attention in the field of drug delivery as carriers since they present a large surface area and can control the release of the drug [26]. Unlike nanoparticles, microparticles are unable to penetrate most biological barriers and remain in the location of interest [27,28,29]. This feature entails an advantage for local delivery of drugs, where systemic absorption is not desired and may result in toxicity issues. For the production of aerogel microparticles, several technologies have been described [30]. The emulsion-gelation method is a suitable option to obtain homogeneous particle sizes, but involves the use of emulsifying agents that may modify the surface properties of the particles [31]. Other approaches are often modifications of the conventional dripping method [30], applying different types of forces (electrostatic, vibrational, or mechanical) to break the liquid jet into droplets. However, the mechanical forces are the only feasible option to process solutions of high viscosity.

**Figure 1:**
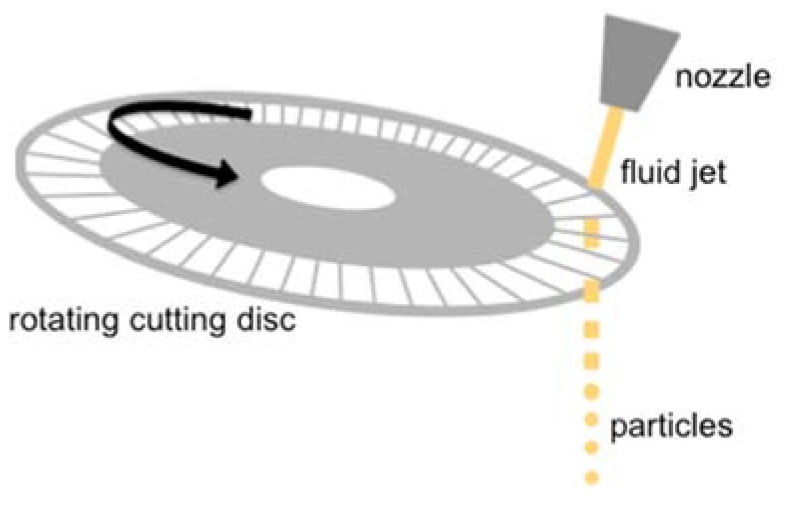
Schematic representation of the jet cutting process. The chitosan solution was pressed out of the nozzle as a fluid jet and cut into cylinders by the cutting disc. The cylinders acquired the spherical shape of droplets before falling into the gelation bath due to surface tension.

The jet cutting method is a simple strategy for the production of gel particles, and is based on the application of mechanical forces to a liquid jet. This technique allows for the preparation of particles with controlled diameters ranging from 100 μm to several millimeters at high production rates [32]. In the jet cutting technique the fluid is pressed out of a nozzle as a jet towards a rotating disc with small wires placed below (Figure 1). This disc cuts the liquid jet into cylinders that would eventually form spherical beads due to surface tension forces. The size of the beads can be modulated by the pressure of the jet, the nozzle diameter, the number and diameter of wires, and the rotation velocity. A small part of the fluid (1–5%) can be lost during the cutting because of its adhesion to the wires and non-zero wire thickness [30]. The adjustment of the parameters to optimize the process along with reuse of the lost solution can lead to production yields close to 100%, making it a very efficient and easy-to-scale process [33].

Overall, chitosan aerogel microparticles are attractive drug carrier candidates for wound treatment, but their processing difficulties, associated with the high viscosity of the chitosan precursor solution, hamper their potential use. Alternative technologies must be sought to overcome the limitations in the processing of chitosan aerogels as microparticles. In this work, the feasibility of the jet cutting technique to obtain chitosan aerogel microparticles was studied, and their potential application in wound treatment was assessed. Chitosan aerogel microparticles were prepared by the sol-gel method using the jet cutting technique followed by supercritical drying with scCO_2_ and evaluated for wound healing purposes. The parameters of the jet cutting process were defined regarding the processability of the chitosan solution and the characteristics of the produced particles. Chitosan particles were loaded with vancomycin HCl, a broad-spectrum antimicrobial drug, and the drug loading and release were evaluated. Specific tests for the application in wound treatment, as the exudate sorption capacity, antimicrobial activity, hemocompatibility, and cytocompatibility, were also carried out.

## 2. Materials and Methods

### 2.1. Materials

Chitosan (degree of deacetylation 90%, viscosity 500 mPa·s, Mw 200–400 kDa) was purchased from Heppe Medical Chitosan GmbH (Halle, Germany). Glacial acetic acid (100% purity) and ethanol (99.8% purity) were obtained from Carl Roth (Karlsruhe, Germany) and CO_2_ (purity > 99.5%) was supplied from Praxair (Ratingen, Germany). NaCl and NH_3_ (25% in H_2_O) were from PanReac AppliChem (Barcelona, Spain). Triton X-100 was from Merck (Darmstadt, Germany). Vancomycin hydrochloride (Mw 1486 g/mol, 94.3% purity, amorphous) was from Guinama (Valencia, Spain). BALB/3T3 clone A31 mouse fibroblasts (ATCC CCL-163) and Dulbecco’s modified Eagle’s medium (DMEM) were from the American Type Culture Collection (ATCC, Manassas, VA, USA). Fetal bovine serum (FBS), phosphate buffer saline (PBS), and penicillin 10,000 U/mL-streptomycin 10 mg/mL, NaOH and HCl 37% were supplied by Sigma-Aldrich (Saint Louis, MO, USA). WST-1 reactive was purchased from Roche (Basel, Switzerland). 

### 2.2. Production of Chitosan Aerogel Microparticles

Chitosan gel particles were produced using a JetCutter Type S equipment (GeniaLab GmbH, Braunschweig, Germany). Table 1 summarizes the combinations of parameters used in this study. Briefly, the jetted chitosan solution (250 mL of 2 wt.% chitosan in 1% v/v acetic acid aqueous solution) was extruded through a nozzle assisted by compressed air (P = 2 bar). A protective piece of stainless steel was placed around the cutting disc to collect fluid loss. Different nozzle diameters (350, 400, and 500 μm), number of wires (40 and 120) of the cutting disc, and cutting disc rates (1000 to 6000 rpm) were used to test the feasibility of particle production and the morphology and particle size distribution (PSD) of the resulting particles. The angle of the jet was in all cases perpendicular to the cutting disc. A gelation bath consisting of 2 L of alkaline medium was placed below the jet cutter to form and collect the gel microparticles. Preliminary tests were carried out in aqueous media (0.2 M NaOH) to avoid the use of high volumes of ethanol and thus reducing the amount of organic solvents used in the study. After a preliminary screening of the parameters for the processing of the chitosan solution, the gelation of vancomycin-loaded chitosan particles was performed in 2 L of EtOH containing 26 mL of 25% aqueous NH_3_. The loading of vancomycin HCl into the particles was performed by addition of the drug to the initial chitosan solution (10 wt.% with respect to chitosan). The microparticles were left in the gelation bath for 1 h for ageing and then the solvent was replaced with absolute EtOH twice. Gel particles were placed in filter paper and dried for 3.5 h with supercritical CO_2_ in a 250 mL high pressure autoclave (120 bar, 40 °C, 15 g/min).

### 2.3. Morphology and Textural Properties

During the initial screening of the jet cutting process, gel particles processed using nozzle diameters of 400 and 500 μm and gelified for 1 h in aqueous 0.2 M NaOH were examined by optical microscopy (VisiScope TL384H, VWR International GmbH, Darmstadt, Germany) to qualitatively monitor properties such as sphericity and homogeneity. After supercritical drying, the resulting unloaded and vancomycin-loaded aerogels were studied by scanning electron microscopy (SEM) at 3 kV (FESEM ULTRA PLUS, Zeiss, Oberkochen, Germany). Prior to SEM-imaging, aerogels were sputtered-coated (Q150 T/S/E/ES, Quorum Technologies, Lewes, UK) with a 10 nm layer of iridium to improve the contrast. The PSD and sphericity of the aerogels were determined by dynamic image analysis (CamSizer XT, Retsch, Haan, Germany). All data for the PSD were obtained based on the x_area_, i.e., the particle diameter obtained from the area of particle projection. Sphericity was given as a value between 0 and 1, with 1 being a perfect sphere. 

Nitrogen adsorption-desorption measurements (ASAP 2000 Micromeritics Inc, Norcross, GA, USA) were used for the determination of the textural properties of the aerogel particles loaded with vancomycin HCl and gelified in a NH_3_/EtOH medium. Specific surface area (a_BET_) was calculated using the Brunauer-Emmet-Teller (BET) method, whereas the Barrett-Joyner-Halenda (BJH) method was applied for the determination of the pore size distribution, specific pore volume (V_p,BJH_), and mean pore diameter (d_p,BJH_). Overall porosity (ε) was determined using Equation (1):

(1) $$\varepsilon =\left(1-\frac{\rho_{\mathrm{bulk}}}{\rho_{\mathrm{skel}}}\right)\times 100$$

where ρ_bulk_ is the bulk density determined from the weight of particles of a known volume, and ρ_skel_ is the skeletal density determined by helium pycnometry (MPY-2, Quantachrome, Delray Beach, FL, USA). 

### 2.4. Fluid Sorption Capacity Test

Approximately 40 mg of aerogel microparticles were placed in 6-well plate inserts of known weight and immersed in Falcon tubes containing 50 mL of PBS (Phosphate Buffered Saline) pH 7.4 solution. At specific times (1, 2, 4, 8, and 24 h), the inserts were removed from the solution and weight gain was determined. The experimental test was carried out in triplicate. The PBS sorption capacity was calculated using Equation (2):

(2) $$\mathrm{PBS}\;\mathrm{sorption}\;\left(\%\right)=\frac{w_{t}-w_{0}}{w_{0}}\times 100$$

where w_0_ and w_t_ are the weight of the particles before and after the immersion in PBS during a certain time t, respectively. 

### 2.5. Vancomycin Entrapment Yield and Release Tests

Vancomycin-loaded chitosan aerogel particles (50 mg) were placed in glass vials containing 5 mL of 0.1 M HCl. After 4 h, the particles were dissolved and the concentration of vancomycin in the medium was determined by UV/Vis spectrophotometry at a wavelength of 280 nm (Genesys10uv, Thermo Spectronic, New York City, NY, USA). The absorbance of dissolved unloaded chitosan aerogels was also determined to remove the influence of the polymer in the UV-measurements. The concentration of vancomycin HCl was calculated using a calibration curve in 0.1 M HCl validated in the 20–300 μg/mL range (R^2^ > 0.9995). The entrapment yield of vancomycin into the aerogels was determined using Equation (3):

(3) $$\mathrm{Entrapment}\;\mathrm{yield}\;\left(\%\right)=\frac{w_{p}}{w_{t}}\times 100$$

where w_p_ is the amount of vancomycin HCl present in the particles and w_t_ is the total amount of vancomycin added to the initial chitosan solution.

Vancomycin release tests were carried out in Franz cells consisting of a donor chamber and a receptor chamber separated by a 0.45 μm cellulose nitrate membrane filter (Whatman, Maidstone, UK). The receptor chamber was filled with 6 mL of PBS (pH 7.4) and *ca.* 40 mg of particles were added to the donor chamber. Surface available for drug diffusion was 1 cm^2^. The release tests were performed in triplicate in an orbital shaker (VWR^®^ Incubating Mini Shaker, VWR, Chester, PA, USA) at 37 °C and 400 rpm. At preset times, aliquots of 1 mL were taken from the receptor chamber and the withdrawn volume was replaced with fresh PBS. The concentration of vancomycin HCl was determined by UV–Vis spectrophotometry (8453, Agilent, Santa Clara, CA, USA) using a calibration curve in PBS validated in the 25–200 μg/mL range (R^2^ > 0.9997). Experiments were carried out in triplicate and results were expressed as μg of vancomycin released per mg of loaded aerogel particles.

### 2.6. Antimicrobial Tests

Antibacterial activity of the aerogel microparticles was tested against *S. aureus* (ATCC 25923). Exponential bacterial culture (10^6^ CFUs/mL) was prepared in a simulated body fluid (SBF, pH 7.4). The bacterial suspension (200 mL) and 7 mg of chitosan aerogel particles (with and without vancomycin) were incubated at 37 °C and 150 rpm for 6, 24, and 48 h. After incubation, the planktonic population was quantified by the colony-forming units (CFUs) method. A solution of vancomycin HCl (1.85 mg/mL) and free bacterial suspension acted as positive and negative controls, respectively. Three independent experiments were performed in triplicate. Results were expressed as the logarithmic concentration of planktonic bacteria.

### 2.7. Biocompatibility Tests in vitro

#### 2.7.1. Hemolytic Activity Test

The hemolytic activity of the vancomycin-loaded aerogel microparticles was tested using human blood (Galician Transfusion Center, Spain) obtained in accordance with the rules of the Declaration of Helsinki. A sample of fresh human whole blood was diluted to 33% (v/v) in 0.9% (w/v) NaCl and 1 mL of the diluted blood was poured in Eppendorf tubes containing 5 mg of vancomycin-loaded chitosan aerogel microparticles, 100 μL of 4% (v/v) Triton X-100 (positive control) or 100 μL of 0.9% (w/v) NaCl (negative control). Samples were incubated for 60 min at 37 °C and 100 rpm in an orbital shaker and then centrifuged at 10,000 g for 10 min (Sigma 2-16P, Sigma Laboratory Centrifuges, Germany). Then, 150 μL of the supernatant were transferred to a 96-well plate and the absorbance of the hemoglobin was measured at 540 nm (FLUOStar Optima, BMG Labtech, Germany). The percentage of hemolysis of the aerogels was determined using Equation (4):

(4) $$\mathrm{Hemolysis}\;\left(\%\right)=\frac{{\mathrm{Abs}}_{s}-{\mathrm{Abs}}_{n}}{{\mathrm{Abs}}_{p}-{\mathrm{Abs}}_{n}}\times 100$$

where Abs_s_ is the absorbance of the samples containing the aerogels, Abs_n_ is the absorbance of the negative control (0% of hemolysis), and Abs_p_ is the absorbance of the positive control (Triton X-100, 100% of hemolysis). Tests were carried out in triplicate.

#### 2.7.2. Cytotoxicity Test

The compatibility of vancomycin-loaded aerogel microparticles was tested against BALB/3T3 mouse fibroblasts. Cells were seeded in 24-well plates (12,350 cells per well) in DMEM supplemented with 10% FBS, penicillin 100 U/mL, and streptomycin 100 μg/mL and incubated overnight at 37 °C in a humidified atmosphere with 5% CO_2_. Then, four replicates of 5 mg of particles were sterilized using UV radiation (30 min, 254 nm), placed in cell culture inserts (Thermo Fisher Scientific, Waltham, MA, USA), and immersed in the wells. Cells cultured without particles were the positive control. After 24 and 48 h of incubation, the inserts with the particles were collected and 50 μL of WST-1 reactive were added to each well. After 4 h of incubation, plates were shaken thoroughly for 1 min and 100 μL from each well were transferred to a 96-well plate in triplicate. The absorbance was measured at 450 nm in a plate reader (EnSpire, PerkinElmer, Madrid, Spain) and cytocompatibility was determined using Equation (5):

(5) $$\mathrm{Cell}\;\mathrm{viability}\;\left(\%\right)=\frac{{\mathrm{Abs}}_{s}}{{\mathrm{Abs}}_{c}}\times 100$$

where Abs_s_ and Abs_c_ are the absorbance of the wells cultured with and without (control) the aerogels, respectively.

## 3. Results and Discussion

### 3.1. Jet Cutting of Chitosan Gels and Morphology and Textural Properties of the Resulting Aerogel Particles

The processability of a viscous chitosan solution with the jet cutter was studied producing gel microparticles under different conditions, using an aqueous basic solution (0.2 M NaOH) as the gelation bath. Chitosan gelation took place immediately after contact of the droplet of chitosan solution with the surface of the gelation bath. The change from an acidic to alcaline medium caused the deprotonation of the amino groups of the chitosan, and thus its gelation by a precipitation mechanism. In general, smaller particle sizes were obtained when using smaller nozzle diameters and a higher number of wires in the cutting disc that cut the fluid jet at a higher frequency. However, the use of the smallest nozzle diameter (350 μm) in this study led to frequent events of clogging. Nozzle diameters of 400–500 μm showed good processability and particles were produced at different cutting disc rates (2000, 4000, and 6000 rpm). The 120-wired cutting disc resulted in high fluid losses since it was not able to split the fluid jet into cylinders and the solution remained attached to the wires until deviated to the collector of fluid loss instead of the gelation bath. The chitosan solution successfully reached the gelation bath when the 40-wired cutting disc was used.

**Figure 2:**
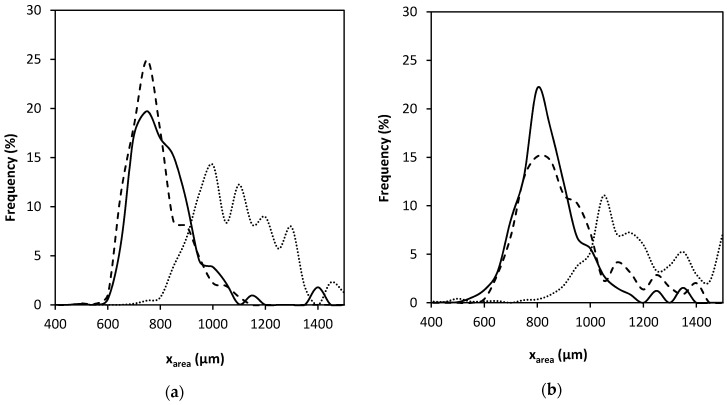
Particle size distribution obtained from the dynamic image analysis of chitosan aerogel particles processed using nozzle diameters of (**a**) 400 and (**b**) 500 μm. Dotted, continuous, and dashed lines represent cutting disc velocities of 2000, 4000, and 6000 rpm, respectively.

**Figure 3:**
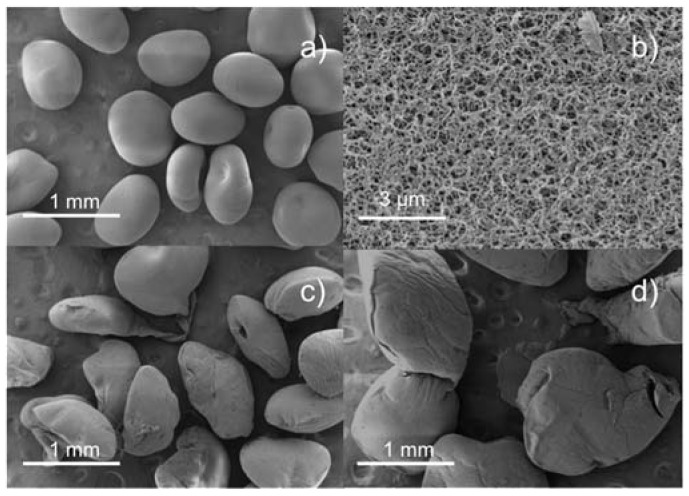
SEM images of chitosan aerogel particles processed with the nozzle diameter of 500 μm at (**a**,**b**) 4000; (**c**) 6000; and (**d**) 2000 rpm.

In accordance with the literature [34], higher nozzle diameters led to larger particle sizes (Table 2 and Figure 2a,b) since the mass flow of the chitosan solution was higher, but similar PSDs were observed using nozzle diameters of 400 or 500 μm. Regarding the cutting disc velocity, aerogels processed at 2000 rpm had larger diameters and broader PSD than those processed at 4000 and 6000 rpm. In general, a higher cutting disc velocity results in smaller particle sizes, but this trend was not herein observed at 4000 and 6000 rpm. This could be explained with the values of sphericity (Table 2) and SEM images of the particles (Figure 3). When using the projected area as the parameter to estimate particle size, if the particle is not spherical the value may be biased by its orientation. Thus, a flattened particle may have the same projected area as a larger spherical particle. Particles processed at 6000 rpm were flatter, probably because of their lower weight and subsequent deformation upon contact with the surface of the gelation medium [35].

The aerogels obtained from chitosan gels processed by jet cutting with a nozzle diameter of 500 μm and a cutting disc velocity of 4000 rpm were lightweight (ρ_bulk_ = 0.060 ± 0.002 g/cm^3^) and highly porous (ε = 95.6% ± 0.2%), and presented excellent textural properties (a_BET_ = 188.0 ± 9.4 m^2^/g). Porosity and bulk density values were consistent with previous reports on chitosan aerogels gelified using a similar precipitation method, but the specific surface area was slightly lower [36,37], probably due to a higher degree of shrinkage of the gel during gelation in ethanol medium [38]. Chitosan aerogels with higher specific surface areas have also been described, but involved the use of chemical crosslinkers that could leave toxic residues in the gels, raising regulatory concerns [39].

### 3.2. Fluid Sorption Capacity

A good moisture balance is required at the wound site for adequate wound epithelization and closure. However, wound exudates are environments rich in inflammatory cytokines and chemokines and can also be a suitable medium for bacterial proliferation [40,41]. Thus, it is important that materials used in wound dressings are able to absorb the exudates, maintaining good conditions for the healing process.

**Figure 4:**
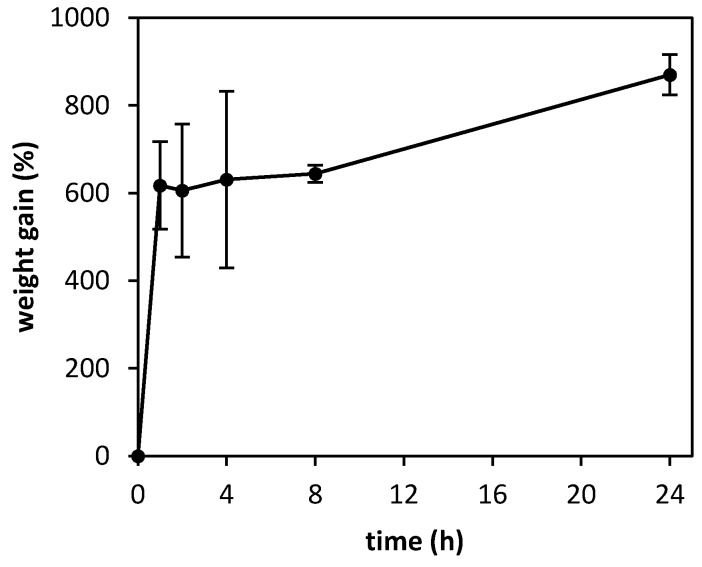
Weight gain after immersion in PBS at 25 °C of chitosan aerogel microparticles processed by a nozzle diameter of 500 μm, a cutting disc velocity of 4000 rpm, and gelified in NH_3_/EtOH solution.

The exudate sorption capacity of the aerogel microparticles was determined by a gravimetric method (Figure 4). Due to their high porosity and large surface area, aerogels were able to absorb up to nine times their weight in PBS after 24 h. Unlike chemical crosslinking, where bonds between the polymer fibers are permanent and may lead to rigid structures with limited water sorption capability [42], the physical precipitation of chitosan allowed for a certain degree of swelling in the polymer network, so the microparticles could retain high amounts of water within their structure.

### 3.3. Drug Loading and Release

Vancomycin HCl is a highly hydrosoluble drug (solubility > 100 mg/mL), but it is poorly soluble in ethanol [43]. Accordingly, chitosan gelation was performed in ethanol with NH_3_ to mitigate drug migration through diffusion to the gelation bath, which would result in low drug- loading efficiencies. The entrapment efficiency for vancomycin contained in the chitosan aerogel microparticles was of 24.6 ± 0.3%, being the final loading in the particles of 22.4 ± 0.3 μg of vancomycin/mg of aerogel particles. The obtained drug loss can be explained by drainage of the water containing the drug from the chitosan solution when dropped in the ethanol of the gelation bath. In any case, the loading was still high if compared to other drug-loaded aerogel formulations prepared in aqueous medium (≈ 12%) [36].

**Figure 5:**
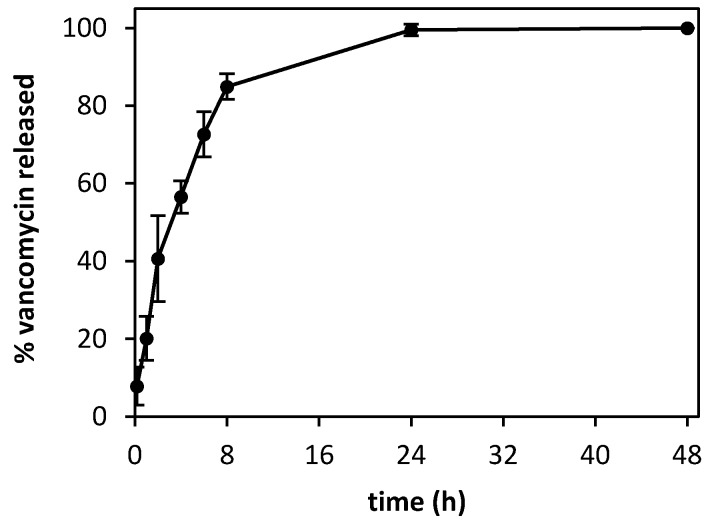
Drug release of vancomycin HCl from the chitosan aerogels (37 °C, 400 rpm, PBS pH 7.4) was sustained over time, reaching 100% of release after 24 h.

In the release studies, aerogel microparticles formed a layer on the membrane of the donor compartment of the Franz cells, simulating their application in the wound. Microparticles only released 50% of the drug payload after 4 h (Figure 5) and complete release was observed after 24 h (the release profile reached a plateau that was kept after 48 h). The microparticles provided concentrations above the MIC (2 μg/mL) for susceptible bacteria already at a short time period, as confirmed in the antimicrobial activity tests (cf. Section 3.4).

### 3.4. Antimicrobial Tests

**Figure 6:**
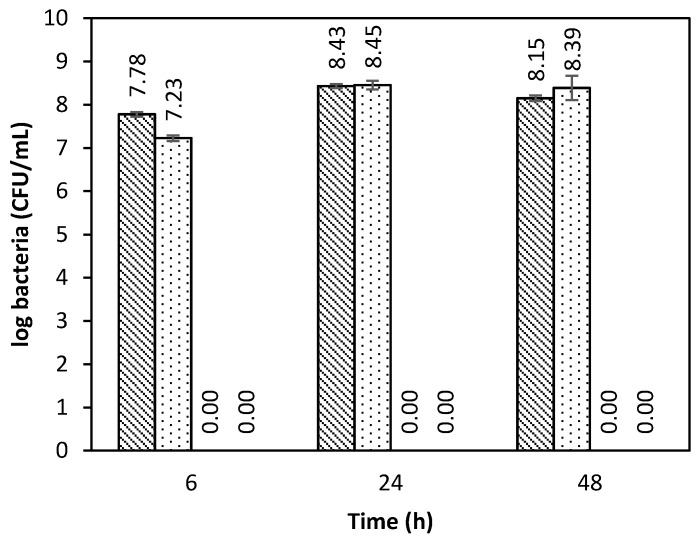
Antimicrobial effect against *S. aureus* strains of vancomycin-loaded chitosan aerogels and dissolved vancomycin HCl (positive control) compared with the negative controls: Free bacterial culture (diagonal bars) and unloaded chitosan aerogels (dotted). Vancomycin in the aerogels and the positive control provided a fast antimicrobial effect, with complete bacterial inhibition after 6 h of incubation.

The antimicrobial activity of the vancomycin-loaded aerogel microspheres was tested in an SBF medium against *S. aureus* (Figure 6), since it is the most common Gram-positive bacteria in chronic wounds [44]. The aerogel microparticles loaded with vancomycin showed a fast antimicrobial effect, being able to completely inhibit the bacterial growth after 6 h of incubation. Bacterial growth inhibition of the vancomycin-loaded aerogels was maintained during the evaluated time (48 h). On the other hand, non-loaded aerogels did not inhibit the bacterial growth.

The antimicrobial effect of the drug-loaded aerogels indicated that the aerogels preserved the active form of vancomycin HCl and released it at an adequate rate. The use of a polymeric matrix that releases the drug instead of the direct use of the drug powder allows for a more precise adjustment of the dosage, avoiding toxic effects [45]. Although many studies have evaluated the antimicrobial capacity of chitosan [46], it has been reported that chitosan only presents antimicrobial activity when dissolved in acidic media [47], probably due to the protonated free amino groups that interfere with the bacterial membrane. 

### 3.5. Biocompatibility and Hemocompatibility of Vancomycin-Loaded Chitosan Aerogel Particles

#### 3.5.1. Hemocompatibility

Biomaterials to be applied directly to the wound need to be compatible with red blood cells, so they do not interfere with the hemostatic activity. A good hemostatic response to the aerogel formulation is crucial, since the first stage of the wound healing process is intended to reduce blood loss and to start the formation of a provisional wound matrix [48]. In later stages of cell proliferation and repair, a process of formation of new blood vessels (angiogenesis) also takes place. The determination of the hemoglobin released from red blood cells from diluted human blood samples after incubation with the material is a simple method to evaluate hemocompatibility. Results showed that the vancomycin-loaded chitosan aerogel microparticles were compatible with the red blood cells compared to the negative control (saline solution), and even the hemolytic activity was lower (−7.7%). According to ISO 10993-4, materials with hemolysis values lower than 5% can be safely used.

#### 3.5.2. Cytocompatibility

**Figure 7:**
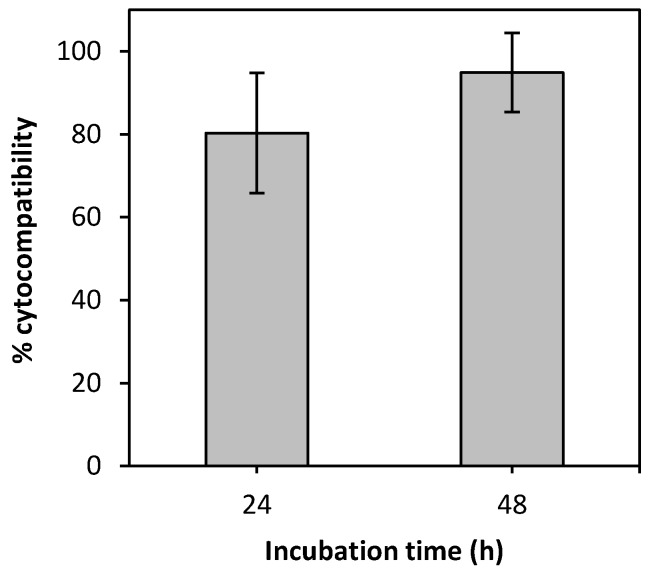
Cytocompatibility of the vancomycin-loaded aerogel microparticles with BALB/3T3 mouse fibroblasts.

Vancomycin HCl is frequently applied by intrawound to prevent post-surgical infection, but it may have a cytotoxic effect at certain concentrations [49]. Therefore, a fibroblast cell line was used to test cell viability after incubation with the vancomycin-loaded chitosan aerogel microparticles (Figure 7). Fibroblasts are the functional cells of the dermis and are responsible for the production of the extracellular matrix, mainly composed of collagen and elastin [50]. During the proliferative stage of the wound healing process, fibroblasts migrate to the wound site and participate in the granulation process by deposition of collagen fibers that will constitute the scar tissue [51]. Overall, the aerogels presented good biocompatibility, with values higher than 80% (after 24 and 48 h).

## 4. Conclusions

The use of the jet cutting technology in combination with supercritical fluid-assisted drying technique represents an excellent strategy for the processing of aerogels from highly viscous precursor solutions. Chitosan aerogels were successfully produced in the form of spherical microparticles through this combined technology, and presented as suitable drug carriers for wound healing applications. Rotation speed and number of wires of the cutting disc along with nozzle diameter were the key parameters for the jet cutting process to obtain spherical and unimodal aerogel particles in the 700–900 µm range. The processing approach presented is compatible with the loading of drugs in the aerogel structure without the involvement of additional steps. The use of ethanol instead of aqueous baths for chitosan gelation turned an attractive strategy for vancomycin loading since the drug entrapment yield in the resulting aerogel particles was significantly improved. The *in vitro* drug release from the chitosan aerogels provided local concentrations of vancomycin able to inhibit the microbial growth of *S. aureus* bacteria in less than 6 h after treatment. High fluid sorption capacity, hemocompatibility, and cytocompatibility with fibroblasts of the chitosan aerogel formulation were suitable for the intended biomedical application. This aerogel-based formulation can meet the requirements to prevent infections for those cases of treatment of chronic wounds shortly after debridement. Vancomycin-loaded aerogel particles can be directly applied at the wound site or included as a component of a multi-layered dressing.

## Figures and Tables

**Table 1:** Experimental jet cutting parameters tested for the processing of chitosan gel particles.

Number of Wires in the Cutting Disc	Gelation Bath	Nozzle Diameter (μm)	Cutting Disc Velocity (rpm)
120	0.2 M NaOH (aq.)	350	4500 1000
40	0.2 M NaOH (aq.)	400	2000 4000 6000
	0.2 M NaOH (aq.)	500	2000 4000 6000
	NH_3_/EtOH		6000

**Table 2:** Particle size distribution of particles (CamSizer^®^ measurements) processed by nozzle diameters of 400 and 500 μm and using 40-wired cutting disc velocities of 2000, 4000, and 6000 rpm.

	400 μm			500 μm		
	2000 rpm	4000 rpm	6000 rpm	2000 rpm	4000 rpm	6000 rpm
x_area_^1^ ± σ (μm)	1105 ± 238	790 ± 130	754 ± 101	1358 ± 393	820 ± 141	877 ± 141
Mean sph ^2^	0.84	0.90	0.89	0.714	0.92	0.84
% sph > 0.9	22	63	51	7	84	8

^1^ Mean particle size (μm) obtained from the projected area. ^2^ Sphericity of the particles.
